# Supplementary material for: UniTVelo: temporally unified RNA velocity reinforces single-cell trajectory inference
Source: Nat Commun. 2022 Nov 3;13:6586. doi: 10.1038/s41467-022-34188-7 (PMC9633790; doi:10.1038/s41467-022-34188-7)
Supplement: Supplementary file 1 — Supplementary Information [file 41467_2022_34188_MOESM1_ESM.pdf]

UniTVelo: temporally unified RNA velocity reinforces single-cell trajectory inference

Mingze Gao[1], Chen Qiao[1], and Yuanhua Huang[1, 2, *]

[1]School of Biomedical Sciences, University of Hong Kong, Hong Kong SAR, China
[2]Department of Statistics and Actuarial Science, University of Hong Kong, Hong Kong SAR, China
[*]Correspondence: yuanhua@hku.hk

Supplementary Tables

Table S1: Ten different datasets are used to validate the proposed RNA velocity inference method, UniTVelo. This table provides manually annotated trajectories as ground truth and unique features of each biological system.

Datasets	Reference	Annotated Trajectory	Unique Features
Pancreas (with cell cycle)	Bergen et al, 2020	Differentiations	Cell cycle & Multi-branching
Pancreas (without cell cycle)	Lange et al, 2020	Differentiations	Multi-branching
Dentate Gyrus	Bergen et al, 2020	OPCs to OLs	Sparse cell types
Retina Development	Kharchenko, 2021	Differentiations	Cell cycle & Multi-branching
scNT-seq	Qiu et al, 2020	Time after stimulation	Major change in early points
Intestinal Organoid	Battich et al, 2020	Stem cell differentiation	Branching
Hindbrain (pons)	La Manno et al, 2018	COPs to NFOLs to MFOLs	M-shape embedding
Mouse Erythroid	Barile et al, 2021	Erythroid maturation	Transcriptional boosting
Human Erythroid	Barile et al, 2021	Erythroid maturation	Transcriptional boosting
Human Bone Marrow	Setty et al, 2019	HSCs & Erythroid maturation	Multi-kinetic rates

Table S2: Number of cycle genes found which are also highly variable genes. We have noticed that both scVelo and Seurat [1] have their own function (relies on a list of cycle genes defined in [2], 43 S genes and 54 G2M genes in total) to assign each cell with a specific cycle phase, G1, S, and G2M. However, this function also marks cells that are not from cycle phase, thus false positives exist, and hard to discriminate between datasets. By setting a proper threshold, number of cycle genes which are highly variable could be a potential way to identify whether a dataset contains cycle and could be a preliminary way to help users choosing the correct mode of UniTVelo.

Datasets	S	G2M
Pancreas (with cell cycle)	22	26
Pancreas	2	3
Dentate Gyrus	4	3
Retina Development (with cell cycle)	35	42
scNT-seq	3	2
Intestinal Organoid	0	0
Hindbrain (pons)	2	6
Mouse Erythroid	15	21
Human Erythroid	0	0
Human Bone Marrow	0	0

Table S3: Performance comparison across datasets between UniTVelo and scVelo. In-cluster Coherence (ICCoh) is used to evaluate the smoothness and consistency of velocity streams within clusters, Methods. The best performance in each metric is highlighted in bold font. Sto: scVelo's stochastic mode. Dyn: scVelo's dynamical mode.

Datasets	scVelo (Sto)	scVelo (Dyn)	UniTVelo
Pancreas (with cell cycle)	0.799	**0.872**	0.632
Pancreas (without cell cycle)	0.769	**0.874**	0.619
Dentate Gyrus	**0.898**	0.859	0.769
Retina Development	**0.933**	0.861	0.666
scNT-seq	0.666	0.838	**0.984**
Intestinal Organoid	0.861	0.804	**0.986**
Hindbrain (pons)	0.896	0.917	**0.990**
Mouse Erythroid	0.713	0.827	**0.991**
Human Erythroid	0.814	0.889	**0.984**
Human Bone Marrow	0.936	0.918	**0.977**

Table S4: Runtime comparison across datasets between scVelo dynamic mode and UniTVelo. Runtime required for each method generally has a positive correlation with number of cells within dataset. Dynamic mode in scVelo has a lower time complexity than UniTVelo possibly because the latter one uses gradient descent during optimization.

Datasets	Number of Cells	Number of Velocity Genes	scVelo (Dyn)	UniTVelo
Pancreas (with cell cycle)	3, 696	1028	1 min 15 s	10 min 17 s
Pancreas (without cell cycle)	2, 531	918	52 s	6 min 41 s
Dentate Gyrus	2, 930	856	33 s	7 min 59 s
Retina Development	2, 726	686	31 s	5 min 24 s
scNT-seq	3, 066	143	14 s	2 min 25 s
Intestinal Organoid	3, 831	414	42 s	4 min 35 s
Hindbrain (pons)	6, 307	637	1 min 04 s	6 min 30 s
Mouse Erythroid	9, 815	503	1 min 29 s	2 min 52 s
Human Erythroid	35, 877	737	9 min 15 s	64 min 16 s
Human Bone Marrow	5, 780	447	3 min 23 s	14 min 28 s

Table S5: Memory usage for UniTVelo and scVelo dynamical mode. The difference of memory usage between two methods are subtle, however, UniTVelo additionally utilizes GPU for model acceleration. All units are measured in GB.

Datasets	UniTVelo (GPU)	UniTVelo (Memory)	scVelo (Memory)
Pancreas (with cell cycle)	1.34	1.38	0.88
Pancreas (without cell cycle)	1.73	1.38	0.88
Dentate Gyrus	1.73	1.38	0.88
Retina Development	1.73	1.25	0.88
scNT-seq	1.66	2.00	0.88
Intestinal Organoid	1.63	2.00	1.00
Hindbrain (pons)	2.72	2.25	1.25
Mouse Erythroid	4.54	2.62	1.62
Human Erythroid	9.06	3.75	4.25
Human Bone Marrow	2.72	3.75	1.88

Supplementary Figures

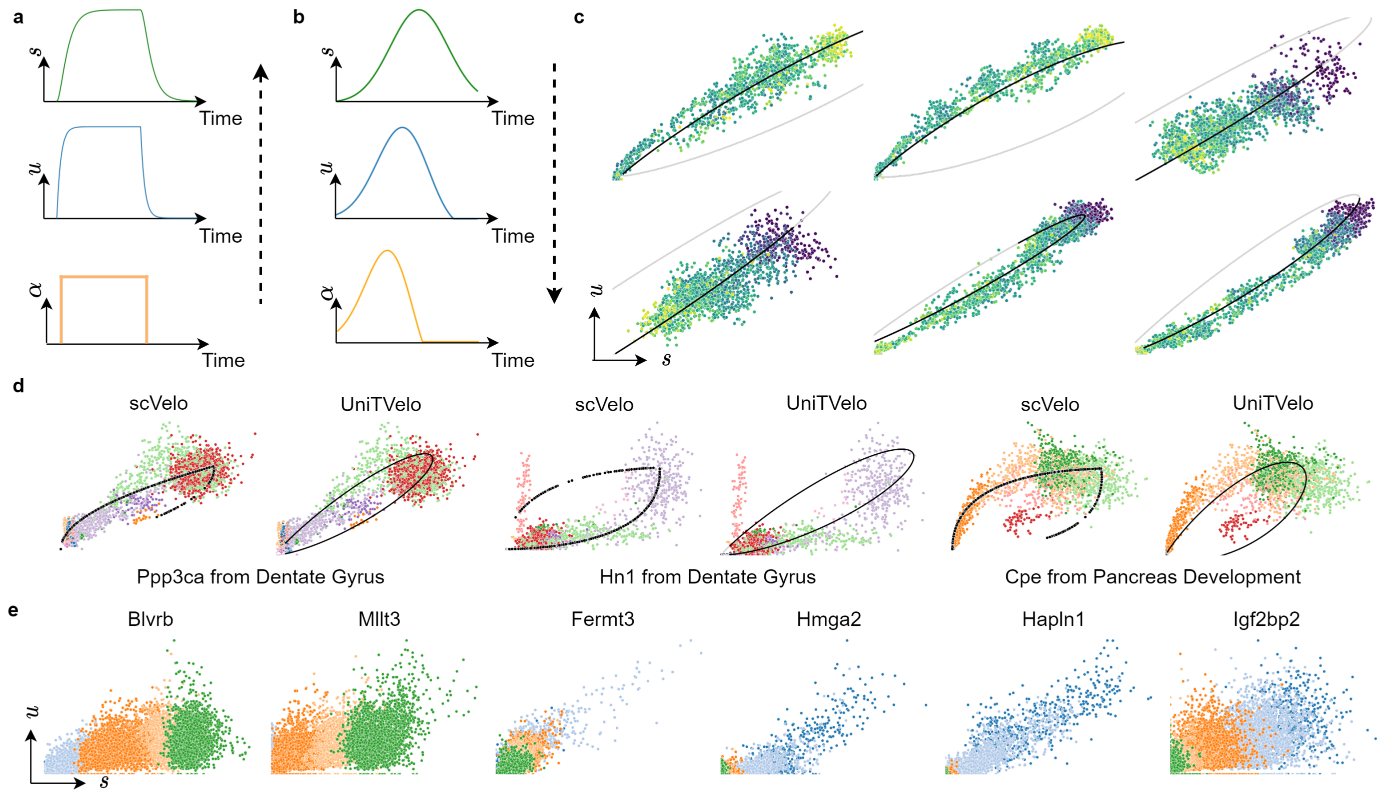

Figure S1: Differences in model design between UniTVelo and scVelo a, Previous framework determine the transcription process often with the order of data generation, e.g. the transcription rate α is defined as step function and other data are derived subsequently. **b,** Top-down strategy adopted by UniTVelo which models the spliced mRNA counts first and other parameters can be derived afterwards. **c,** This top-down design is capable of fitting simulated data generated by first-order dynamics in a bottom-up generation with a step function for transcription rate. **d,** Model used by UniTVelo could have a similar yet smoother relationship between un/spliced counts compared with scVelo regarding to dynamical genes. **e,** A few examples on stably and monotonically changed genes of which expressions change along with time but are generally within steady state. Colors represents different celltypes within lineage and are consistent with Fig.2a.

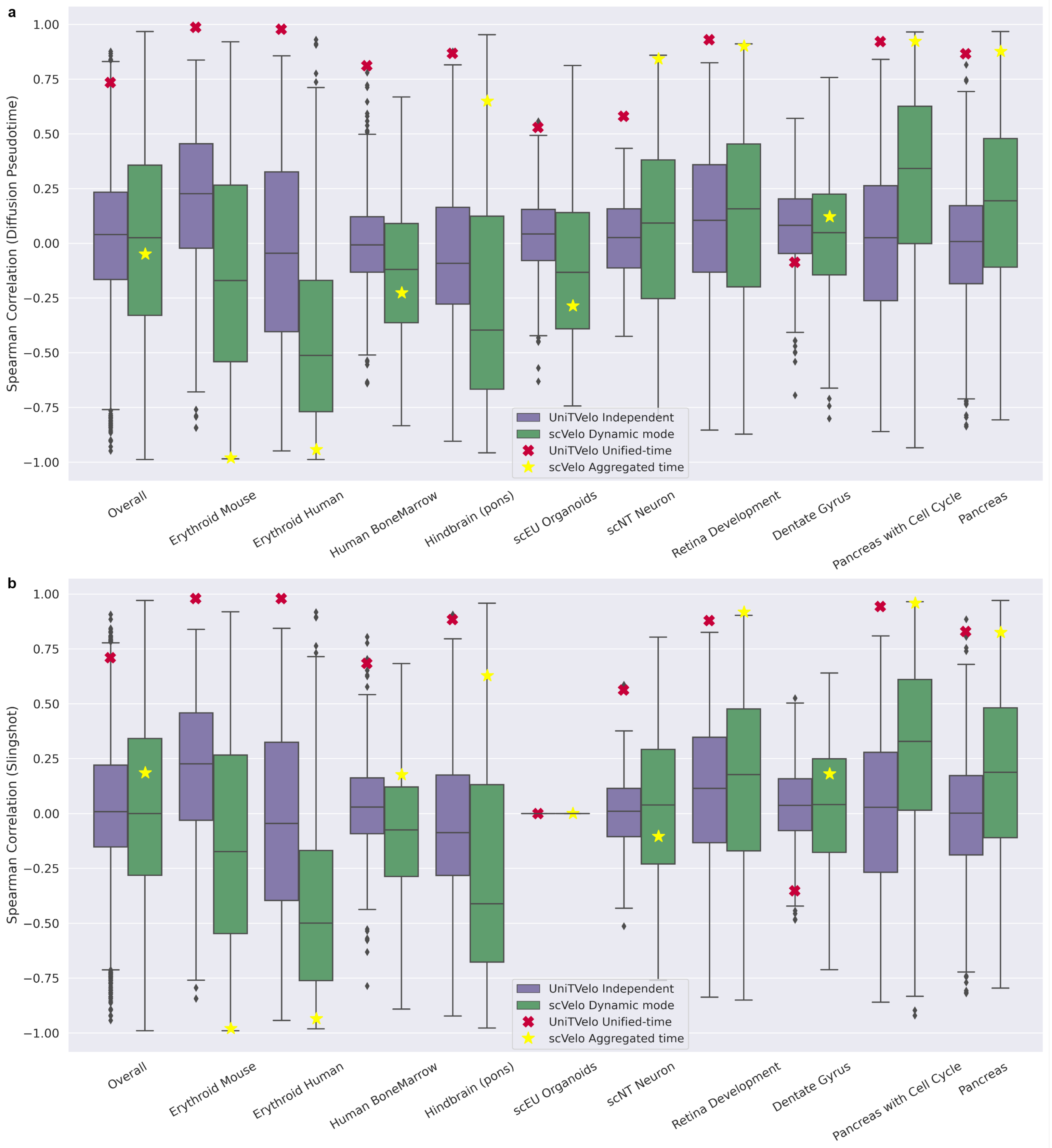
a
Spearman Correlation (Diffusion Pseudotime)
1.00
0.75
0.50
0.25
0.00
−0.25
−0.50
−0.75
−1.00
UniTVelo Independent
scVelo Dynamic mode
UniTVelo Unified-time
scVelo Aggregated time
Overall
Erythroid Mouse
Erythroid Human
Human BoneMarrow
Hindbrain (pons)
scEU Organoids
scNT Neuron
Retina Development
Dentate Gyrus
Pancreas with Cell Cycle
Pancreas
b
Spearman Correlation (Slingshot)
1.00
0.75
0.50
0.25
0.00
−0.25
−0.50
−0.75
−1.00
UniTVelo Independent
scVelo Dynamic mode
UniTVelo Unified-time
scVelo Aggregated time
Overall
Erythroid Mouse
Erythroid Human
Human BoneMarrow
Hindbrain (pons)
scEU Organoids
scNT Neuron
Retina Development
Dentate Gyrus
Pancreas with Cell Cycle
Pancreas

Figure S2: Systematic comparison of two strategies on entire transcriptome. The independent mode of UniTVelo, aka the top-down strategy, fits each gene individually which shares similar logic with dynamical mode of scVelo, the bottom-up method. To directly compare the expression profile fitness of these two strategies, we use diffusion pseudotime and slingshot as reference and calculate the spearman correlation with the assigned gene-specific time matrix across the entire transcriptome. Although the difference of overall performance for 10 datasets shown is subtle between UniTVelo and scVelo, the comparison is done within the independent mode. We also assessed the UniTVelo unified mode (red cross) and the scVelo aggregated latent time (yellow star), where UniTVelo generally performs better than scVelo both in individual datasets or overall performance. The sample size used to derive statistics for each experiment can be accessed through Supplementary Table S4. Data are presented as median values with lower and upper boundaries 25th and 75th percentiles, respectively. Lower and upper error lines represents minima and maxima whilst dots are outliers. **a,** UniTVelo's strategy has better performance than scVelo in 6 datasets (Erythroid Mouse, Erythroid Human, Human BoneMarrow, Hindbrain, scEU Organoids and DentateGyrus), former 5 of which have been demonstrated with superior performance with unified-time mode compared with scVelo, the only exception is the scNT dataset. **b,** Slingshot shows similar results. Slingshot failed to infer a reasonable pseudo-time ordering / trajectory on scEU organoid dataset thus, the spearman correlation is not calculated. Note, here the assessment is based on the whole cell population while the CBDir metric is based on specific cell types, e.g., in the Dentate gyrus datasets.

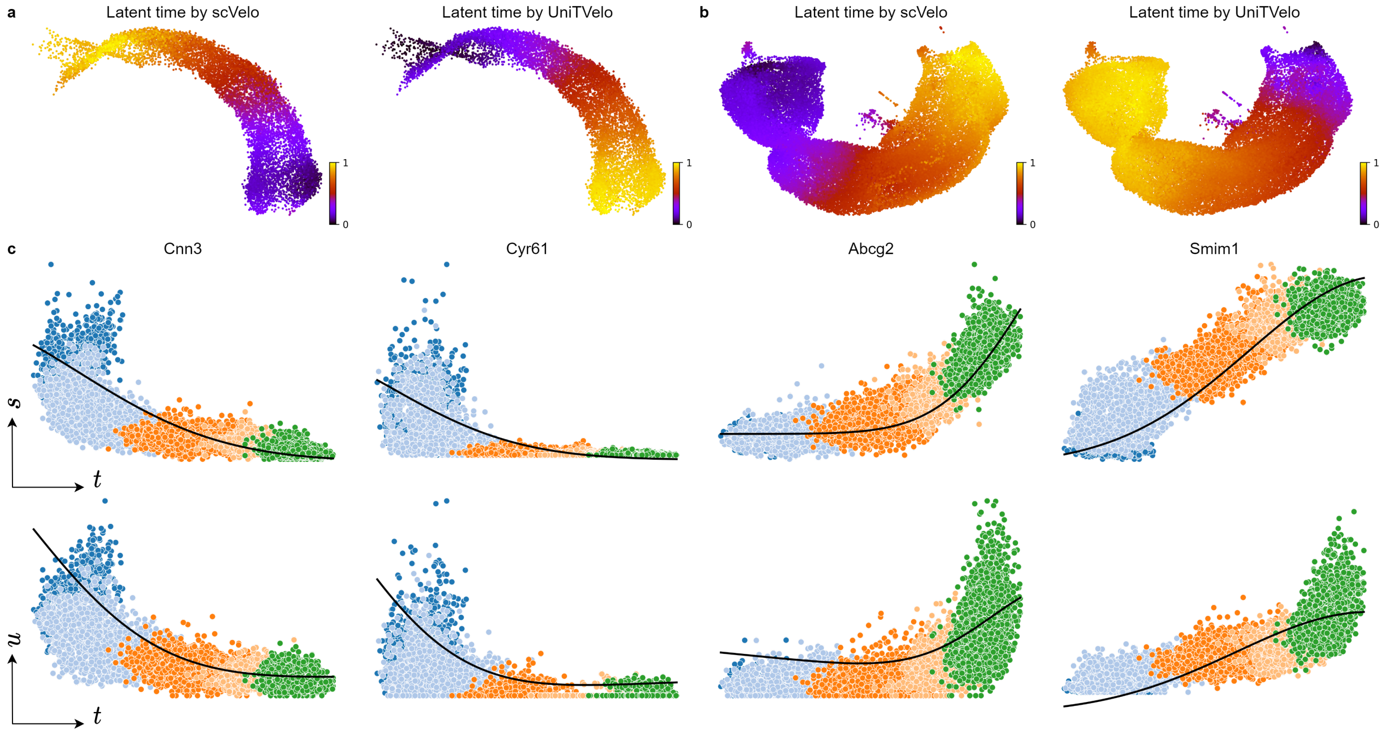

Figure S3: Estimated latent time and transcription activities for mouse and human erythroid maturation. a, Latent time estimated by scVelo (left) and UniTVelo (right) for mouse erythroid lineage. **b,** scVelo (left) returns erroneous temporal information compared with UniTVelo (right) on human erythriod data. **c,** Scatter plot of expression counts illustrates a clear inhibiting behavior for *Cnn3* and *Cyr61* and opposite for *Abcg2* and *Smim1*. Notably, it confirms transcriptional boosting exists in later erythroid lineage, that genes been activated significantly. Black line is model regression result. Various colors differ in celltype and are consistent with Fig.2a.

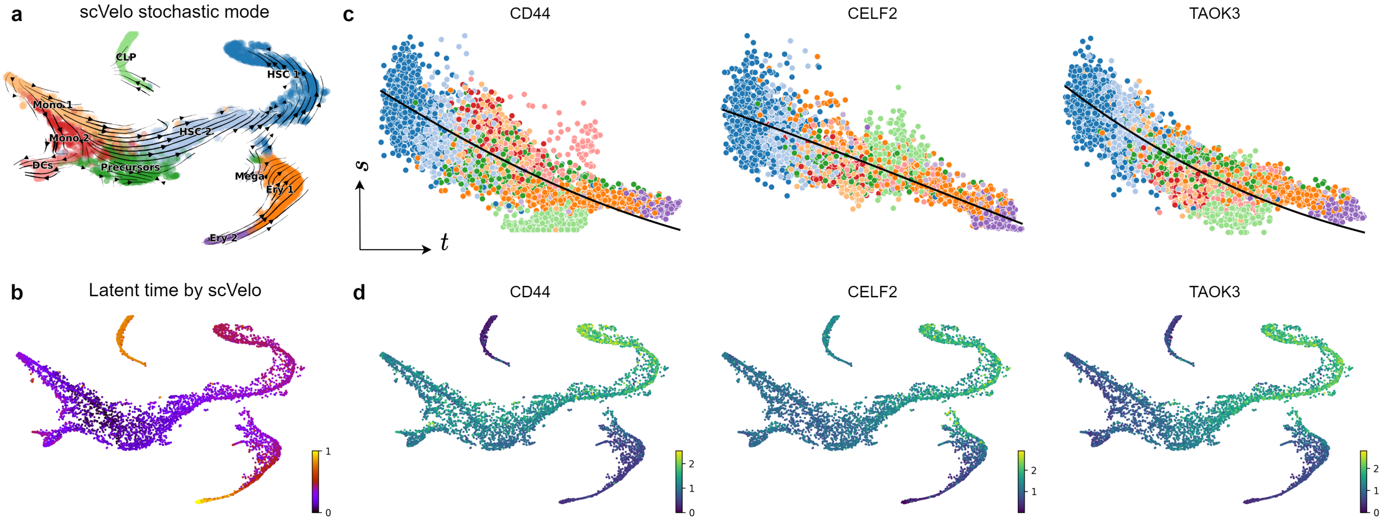

Figure S4: Supplementary information for human bone marrow differentiation analysis. a, scVelo's stochastic mode generates reversed velocity stream in erythroid lineage and distorted velocity fields in both monocytes and CLP lineages. **b,** Latent time estimation by scVelo dynamic mode suggests contradictory time assignments between monocytes and HSCs. **c,** The transcriptome changes along inferred cell time of three example genes shown in Fig.3c suggests they are inhibited during differentiation. Black line is the mean prediction level of transcriptome estimated by UniTVelo. **d,** Expression profiles of relevant genes are in accordance with **c**.

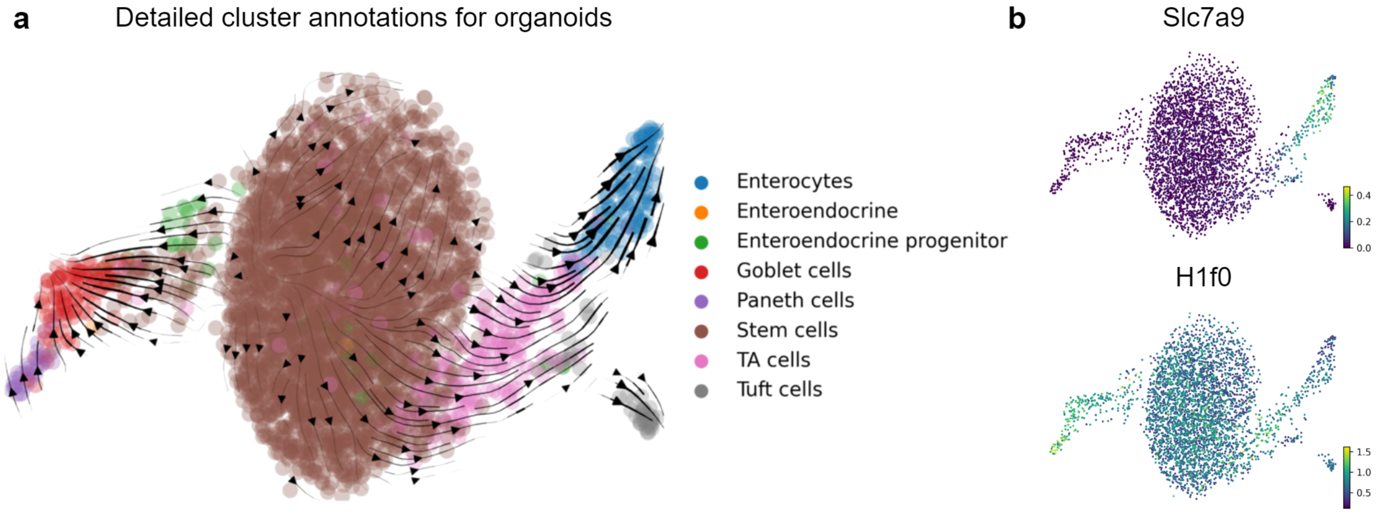

Figure S5: Detailed annotations for intestinal organoids validates correct trajectory by UniTVelo. a, Beside two terminal states, enterocytes and goblet cells at the end of branch, transitions in local regions can also be captured by UniTVelo, e.g. paneth cells are pointing at goblet cells. **b,** Additional examples of expression from *Slc7a9* and *H1f0* are relevant with R^2, suggesting low R^2 genes are less informative.

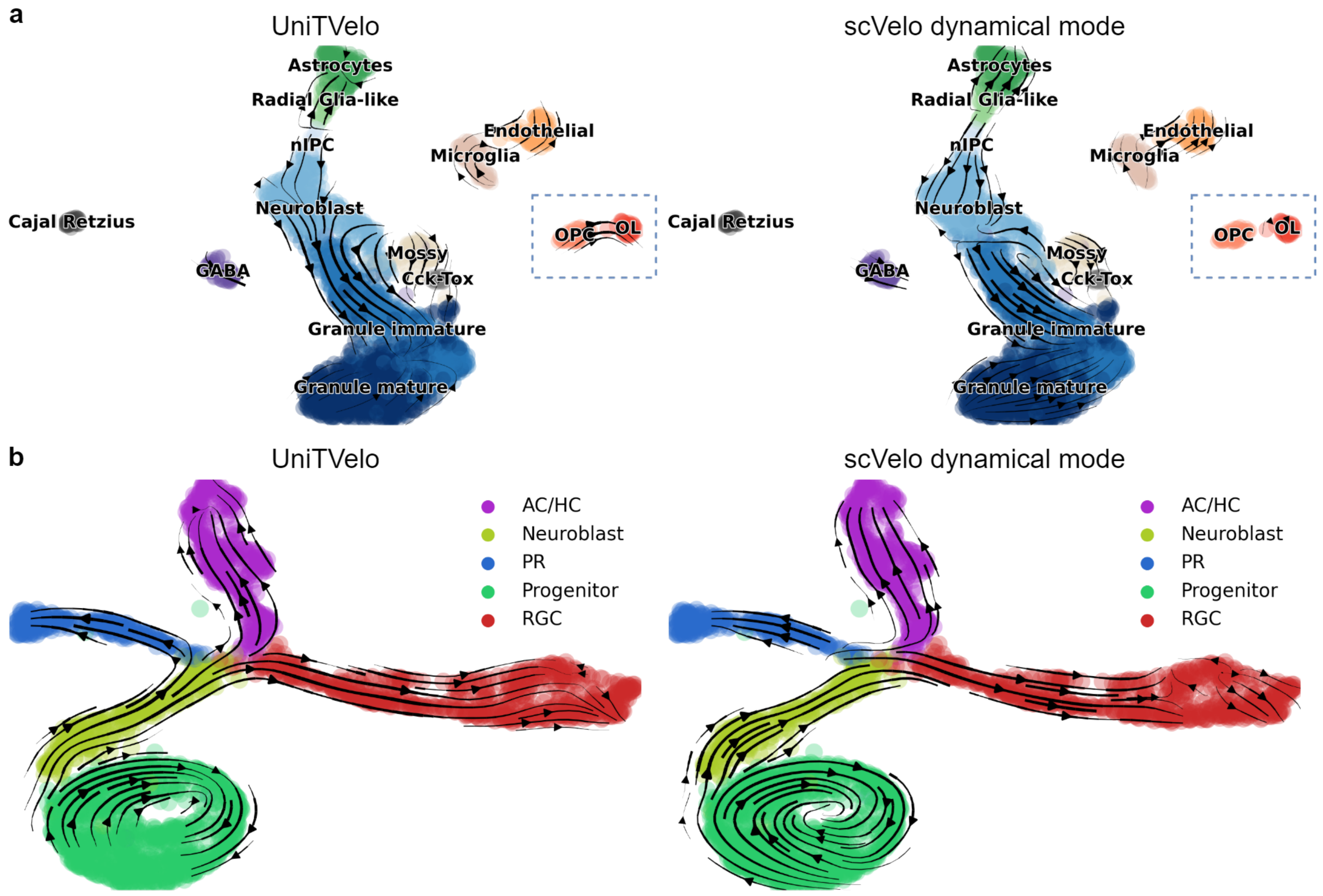

Figure S6: UniTVelo has refined directionality estimation in local regions in intricate differentiation topology. a, Neuron genesis development dataset contains sparse cell types with around 3k cells. Specifically, the development of oligodendrocytes was identified by original paper and UniTVelo inferred a strong directionality between OPCs and OLs (left) whilst this characteristic is less obvious in scVelo dynamical mode (right). **b,** Mouse retinal development dataset contains two topology features, cell cycle and multi-branching. UniTVelo generally produce comparable results in capturing comprehensive features as scVelo and has the ability to refine local regions where velocity fields are not completely in order (the terminal region of RGC branch).

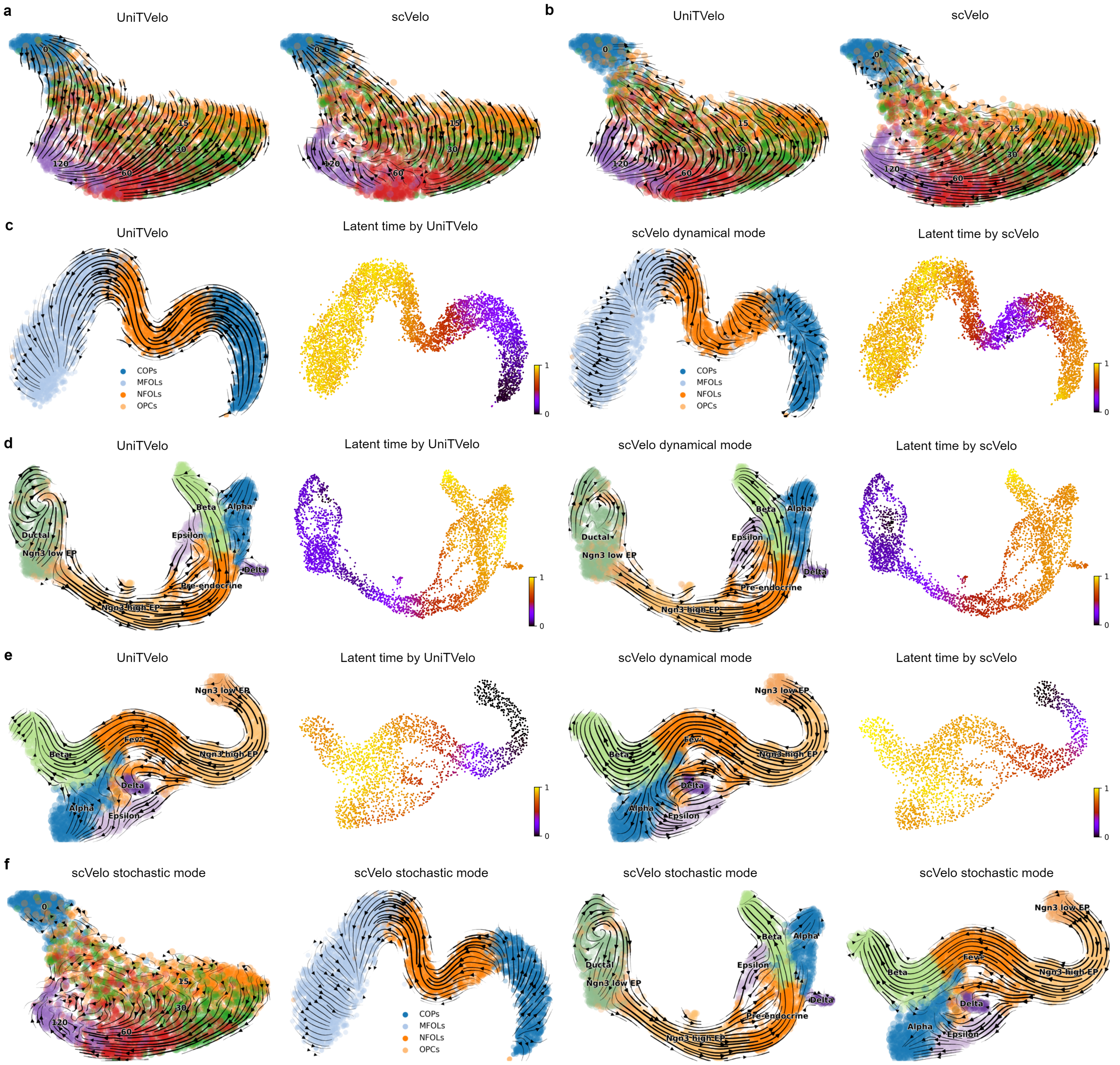

Figure S7: UniTVelo's ability to infer directed trajectories and latent time is further validated on four datasets. **a,** Both scVelo and UniTVelo identified the right direction of neuron development along the stimulation time when using BRIE2 detected differential momentum genes. **b,** When only using highly variable genes selected by scVelo, UniTVelo can preserve the expected direction robustly whilst scVelo could not. **c,** scVelo suffers to find the full differentiation trajectory in the hindbrain (pons) of adolescent mice, whilst UniTVelo managed to identify a continuous path from precursors to matured cells. **d-e,** UniTVelo (the independent mode) and scVelo has comparable performance on major trajectories, e.g. the cycling part or terminal areas. UniTVelo suggests subtle pattern from pre-endocrine (Fev+) to Delta cluster. **f,** Results of scVelo's stochastic mode of the above mentioned datasets are also presented.

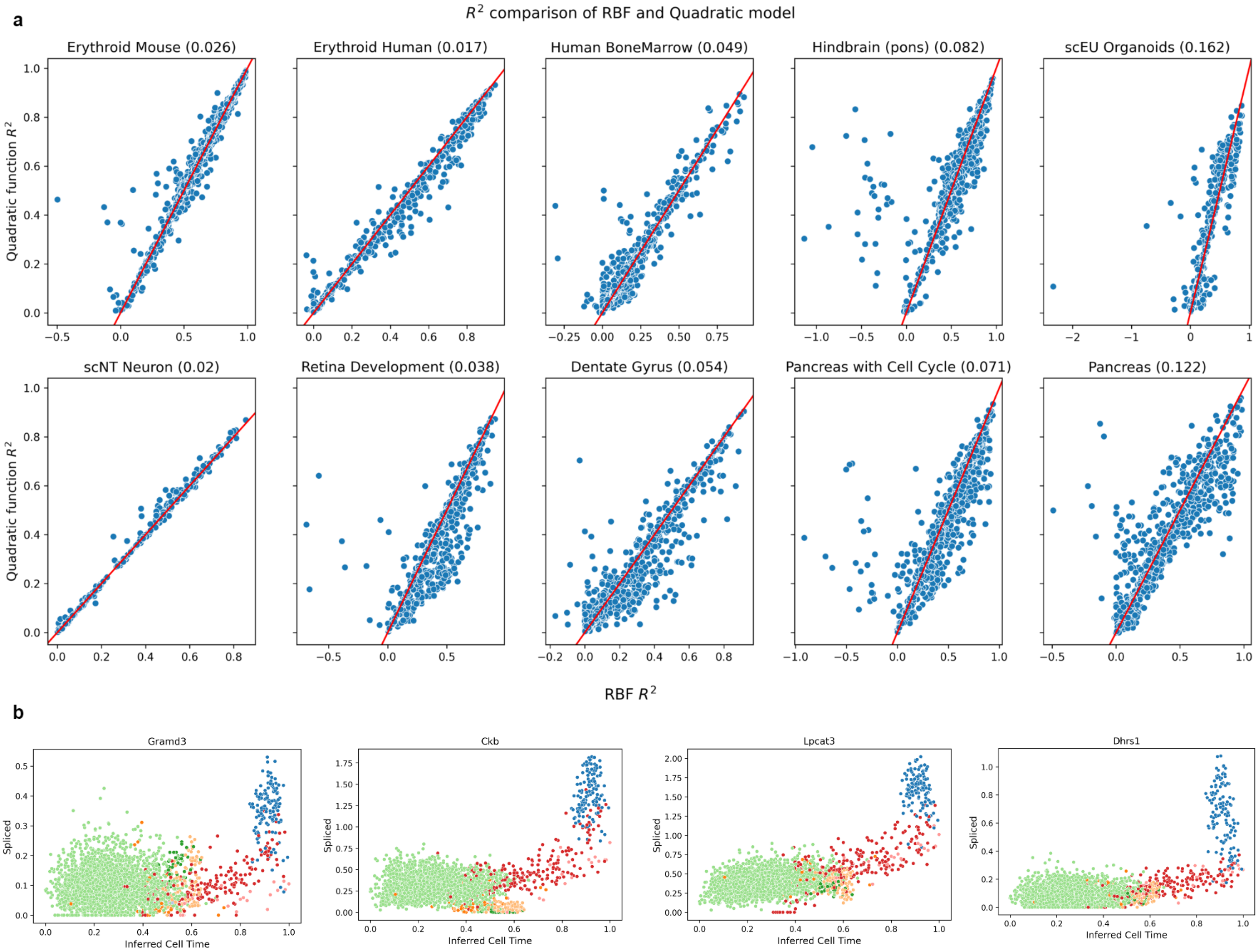

Figure S8: Percentage of reversed transient genes across the entire transtriptome. a, Reversed transient genes are defined as genes with expression profile goes down and up along with cell time. It might be difficult for RBF to model given the convex nature of the shape. To test the hypothesis that whether the abovementioned genes exist and to what extent those genes affect the overall directionality of the velocity field, we have used quadratic function to model the expression profiles of each gene along with inferred cell time and compared the performance with standard RBF function (without unspliced reads). If the transient status of down and up exists, then the parabola should open upwards (positive coefficient) and have a R^2 at least slightly higher than that of RBF model. The above figure shows genes with reversed transient status are quite rare compared to the total number of genes used to construct the velocity graph, normally less than 10%. The numbers behind title are the ratio of identified reversed genes divided by the total number of genes. Note that the threshold to classify whether a gene is reversed transient or not is low (R^2 of quadratic - R^2 of RBF > 0.075) and that results in false positives, but still the ratio is relatively small. We anticipate a smaller ratio if a more stringent threshold is used. **b,** Here are 4 example genes from scEU Organoids dataset which are identified as reverse transient whilst they are not (colors represent different cell clusters).

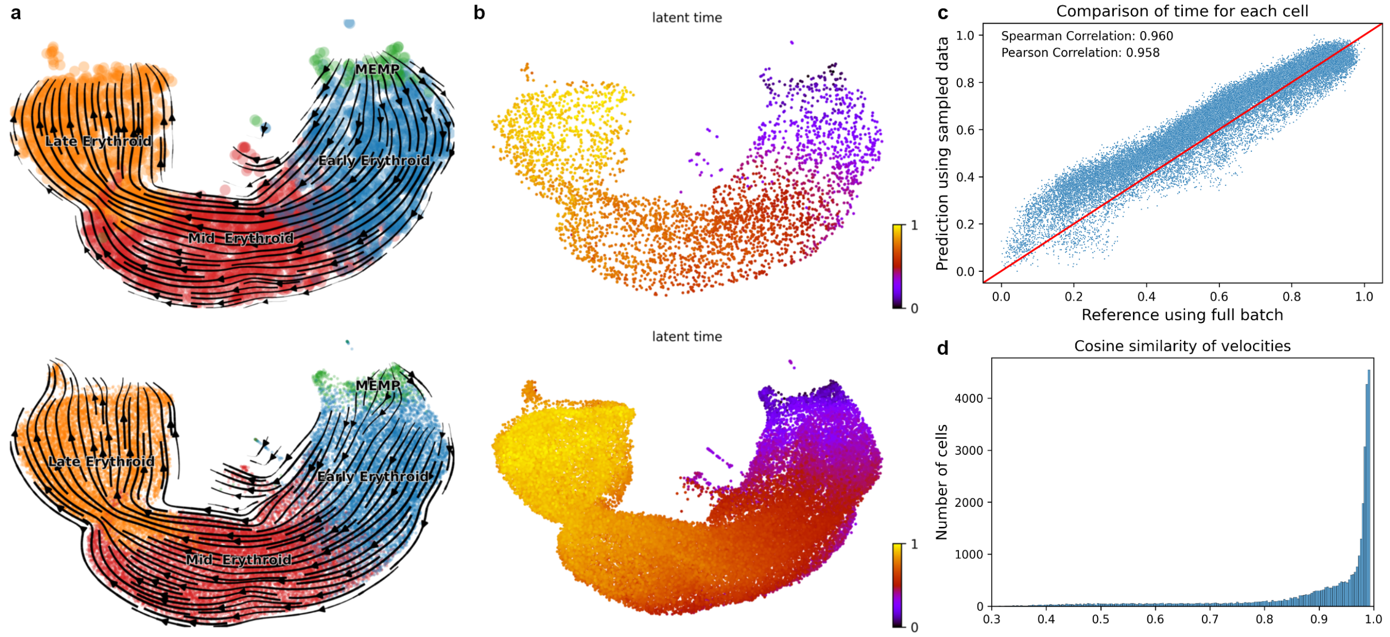

Figure S9: Down-sampling / Prediction strategy on large-scale datasets. Here, we use the erythroid human dataset as an example and briefly describe the results. This dataset originally has 37k cells which use approximately 1 hour to run the model with GPU acceleration. We first randomly sampled 10% of total cells, then the RNA velocity and unified time are inferred based on this subset of data (upper panel of **a** and **b**). Then we use generated parameters to predict the relevant RNA velocity and unified time for the rest of cells (lower panel of **a** and **b**). To assess the performance of this down-sampling / prediction strategy with ground truth (model fitted with full batch of data), we tried two folds of comparison, **c,** The inferred time for each cell is compared between prediction and full batch, the scatter plot showed a strong correlation status. **d,** For RNA velocities for cells, the cosine similarity between prediction and full batch at a high dimensional level is compared. The histogram generally showed a high similarity score which further consolidates our down-sampling / prediction is robust to dense datasets.

References

[1] Hao, Y. *et al.* Integrated analysis of multimodal single-cell data. *Cell* **184**, 3573–3587 (2021).

[2] Tirosh, I. *et al.* Dissecting the multicellular ecosystem of metastatic melanoma by single-cell rna-seq. *Science* **352**, 189–196 (2016).
