# Supplementary material for: UniTVelo: temporally unified RNA velocity reinforces single-cell trajectory inference
Source: Nat Commun. 2022 Nov 3;13:6586. doi: 10.1038/s41467-022-34188-7 (PMC9633790; doi:10.1038/s41467-022-34188-7)
Supplement: Supplementary file 3 — Reporting Summary [file 41467_2022_34188_MOESM3_ESM.pdf]

Corresponding author(s): Yuanhua Huang
Last updated by author(s): Sep 26, 2022

Reporting Summary

Nature Portfolio wishes to improve the reproducibility of the work that we publish. This form provides structure for consistency and transparency in reporting. For further information on Nature Portfolio policies, see our Editorial Policies and the Editorial Policy Checklist.

Statistics

For all statistical analyses, confirm that the following items are present in the figure legend, table legend, main text, or Methods section.

n/a	Confirmed	
☐	☒	The exact sample size (*n*) for each experimental group/condition, given as a discrete number and unit of measurement
☐	☒	A statement on whether measurements were taken from distinct samples or whether the same sample was measured repeatedly
☐	☒	The statistical test(s) used AND whether they are one- or two-sided<br>*Only common tests should be described solely by name; describe more complex techniques in the Methods section.*
☐	☒	A description of all covariates tested
☐	☒	A description of any assumptions or corrections, such as tests of normality and adjustment for multiple comparisons
☐	☒	A full description of the statistical parameters including central tendency (e.g. means) or other basic estimates (e.g. regression coefficient) AND variation (e.g. standard deviation) or associated estimates of uncertainty (e.g. confidence intervals)
☐	☒	For null hypothesis testing, the test statistic (e.g. *F*, *t*, *r*) with confidence intervals, effect sizes, degrees of freedom and *P* value noted<br>*Give P values as exact values whenever suitable.*
☒	☐	For Bayesian analysis, information on the choice of priors and Markov chain Monte Carlo settings
☒	☐	For hierarchical and complex designs, identification of the appropriate level for tests and full reporting of outcomes
☐	☒	Estimates of effect sizes (e.g. Cohen's *d*, Pearson's *r*), indicating how they were calculated

Our web collection on statistics for biologists contains articles on many of the points above.

Software and code

Policy information about availability of computer code

Data collection	Source data are public accessible, processed data. Some are acquired from scVelo 0.2.3
Data analysis	UniTVelo 0.1.6 (https://github.com/StatBiomed/UniTVelo), scVelo 0.2.3, Slingshot 1.8.0, Python 3.8.13

For manuscripts utilizing custom algorithms or software that are central to the research but not yet described in published literature, software must be made available to editors and reviewers. We strongly encourage code deposition in a community repository (e.g. GitHub). See the Nature Portfolio guidelines for submitting code & software for further information.

Data

Policy information about availability of data

All manuscripts must include a data availability statement. This statement should provide the following information, where applicable:

- Accession codes, unique identifiers, or web links for publicly available datasets
- A description of any restrictions on data availability
- For clinical datasets or third party data, please ensure that the statement adheres to our policy

Mouse and human erythroid differentiation
Erythroid lineage which derived from human and mouse gastrulation process. Cells are sequenced using 10X Genomics V2 sequencing protocol based on drop-let method. Human erythroid lineage is available from "Coordinated changes in gene expression kinetics underlie both mouse and human erythroid maturation" whilst mouse gastrulation subset is incorporated by scv.datasets.gastrulation_erythroid()

Human bone marrow hematopoieses
The raw data counts as well as associated experimental details can be accessed through the Human Hematopoietic Profiling project. Processed data is integrated in scVelo via scv.datasets.bonemarrow()

Intestinal organoid differentiation
Datasets have been deposited in GEO with accession number GSE128365. Data and labels used for RNA velocity analysis is available upon requesting

Dentate gyrus neurogenesis development
Experiment of Dentate Gyrus development comprises two time points (P12 and P35) using droplet-based scRNA-seq protocol. Details can be accessed by scv.datasets.dentategyrus()

Mouse developing retina
Raw data of mouse developing retina was sequenced by 10x Chromium and has been deposited in GEO under GSM3466902. Processed data for velocity analysis and result reproduction is downlowded from Kharchenko Lab.

Neuron genesis with KCl stimulation.
Sequenced data within this study is available in Gene Expression Omnibus (GEO) under accession number GSE141851. Processed counts and annotations is available upon requesting.

Hindbrain (pons) of adolescent mice
The differentiation of oligodendrocyte lineage and its associated myelination process is demonstrated. Counts metrics and annotations in .rds format are acquired from Kharchenko Lab.

Pancreatic endocrinogenesis
Pancreatic epithelial cells were sampled at day 15.5 from embryonic with four possible terminal states. Processed data is acquired from scv.datasets.pancreas()

Human research participants

Policy information about studies involving human research participants and Sex and Gender in Research.

Reporting on sex and gender	Research does not contain any human participants
Population characteristics	N/A
Recruitment	N/A
Ethics oversight	N/A

Note that full information on the approval of the study protocol must also be provided in the manuscript.

Field-specific reporting

Please select the one below that is the best fit for your research. If you are not sure, read the appropriate sections before making your selection.

☒ Life sciences ☐ Behavioural & social sciences ☐ Ecological, evolutionary & environmental sciences

For a reference copy of the document with all sections, see nature.com/documents/nr-reporting-summary-flat.pdf

Life sciences study design

All studies must disclose on these points even when the disclosure is negative.

Sample size	In velocity analysis, no sample size calculation was performed in cell-level as all cells from the processed data were used in the model. Genes are selected based on utv.velocity.get_velo_genes function, to filter out genes with extremely low expression level or with inexplicit kinetic rates.
Data exclusions	No data were excluded
Replication	Reproducibility can be achieved following the notebooks provided in GitHub repository
Randomization	Not relevant to the study as this is a computational tool development research, mainly focused on pattern discovery instead of hypothesis testing
Blinding	Not relevant to the study as this is a computational tool development research, mainly focused on pattern discovery instead of hypothesis testing

Reporting for specific materials, systems and methods

We require information from authors about some types of materials, experimental systems and methods used in many studies. Here, indicate whether each material, system or method listed is relevant to your study. If you are not sure if a list item applies to your research, read the appropriate section before selecting a response.

Materials & experimental systems

n/a	Involved in the study
☒	☐ Antibodies
☒	☐ Eukaryotic cell lines
☒	☐ Palaeontology and archaeology
☒	☐ Animals and other organisms
☒	☐ Clinical data
☒	☐ Dual use research of concern

Methods

n/a	Involved in the study
☒	☐ ChIP-seq
☒	☐ Flow cytometry
☒	☐ MRI-based neuroimaging
